# Supplementary material for: Nociceptors use multiple neurotransmitters to drive pain
Source: bioRxiv. 2025 Sep 11:2025.09.09.675093. Preprint. [Version 2] doi: 10.1101/2025.09.09.675093 (PMC12439995; doi:10.1101/2025.09.09.675093)
Supplement: 1 [file NIHPP2025.09.09.675093v2-supplement-1.pdf]

bioRxiv preprint doi: https://doi.org/10.1101/2025.09.09.675093; this version posted September 11, 2025. The copyright holder for this preprint (which was not certified by peer review) is the author/funder. This article is a US Government work. It is not subject to copyright under 17 USC 105 and is also made available for use under a CC0 license.

Supplementary Figures

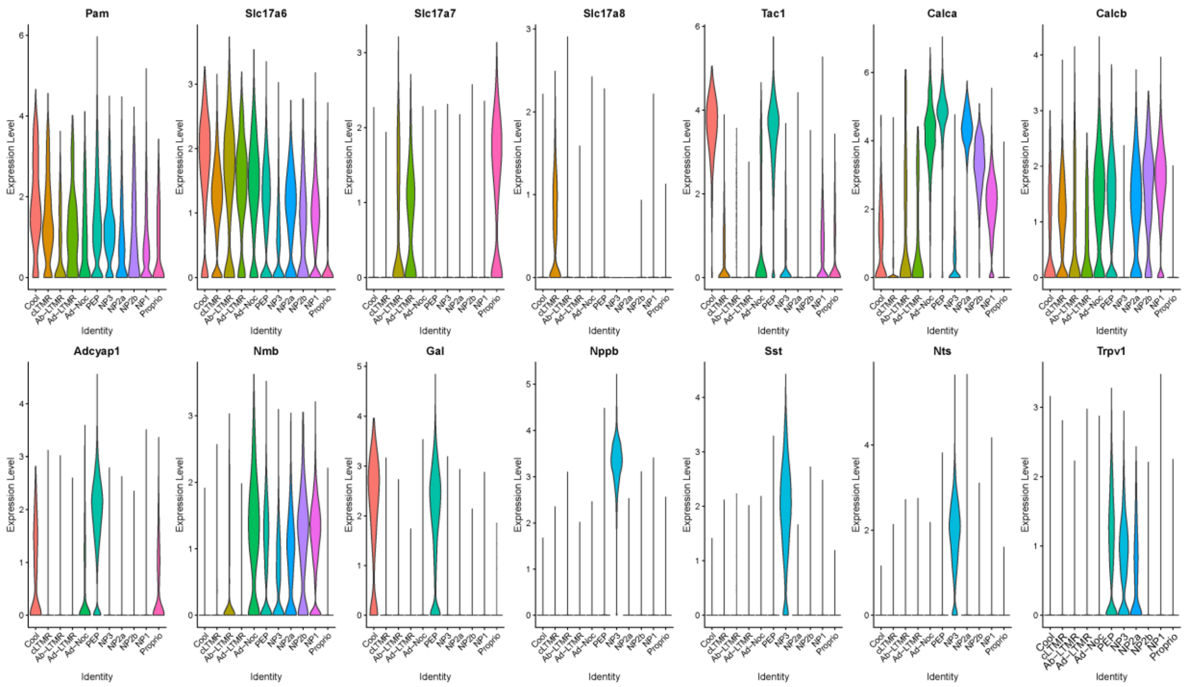

Fig. S1. Subset-specific expression of neurotransmitter and neuropeptide-related genes in mouse dorsal root ganglia neuron subtypes. Violin-plot analysis of expression level (log normalized single cell RNA sequencing data) *(49)* of neuropeptide- and neurotransmitter-related genes across the 10 sensory neuron transcriptomic classes used in Ref. *(50)*.

bioRxiv preprint doi: https://doi.org/10.1101/2025.09.09.675093; this version posted September 11, 2025. The copyright holder for this preprint (which was not certified by peer review) is the author/funder. This article is a US Government work. It is not subject to copyright under 17 USC 105 and is also made available for use under a CC0 license.

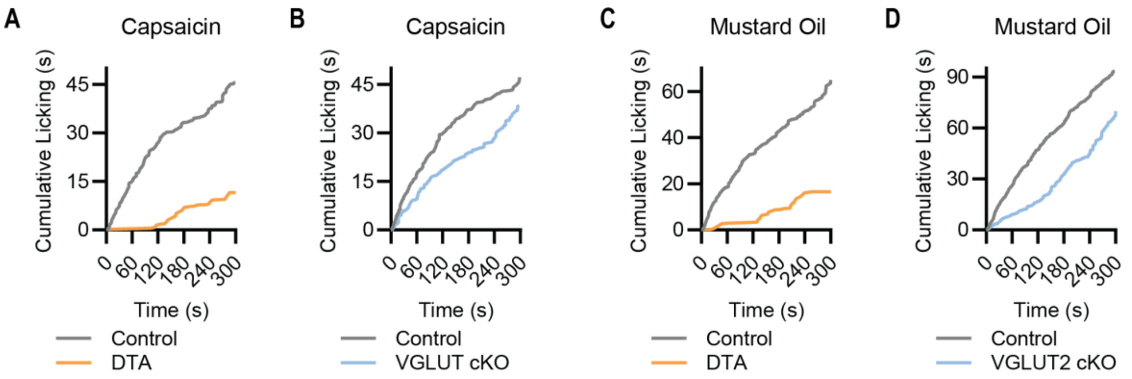

Fig. S2. Temporal structure of chemical pain in DTA and VGLUT2 cKO mice. (A-D) Cumulative paw licking (normalized to number of mice per group) following capsaicin or mustard oil injection in DTA mice, VGLUT2 cKO mice, and their respective littermate controls. For capsaicin, (A) n=9 (5M/4F) for Control and n=5 (4M/1F) for DTA; (B) n=5 (3M/2F) for Control and n=7 (5M/2F) for VGLUT2 cKO. For mustard oil, (C) n=9 (5M/4F) for Control and n=5 (4M/1F) for DTA; (D) n=10 (3M/7F) for Control and n=12 (5M/7F) for VGLUT2 cKO.

bioRxiv preprint doi: https://doi.org/10.1101/2025.09.09.675093; this version posted September 11, 2025. The copyright holder for this preprint (which was not certified by peer review) is the author/funder. This article is a US Government work. It is not subject to copyright under 17 USC 105 and is also made available for use under a CC0 license.

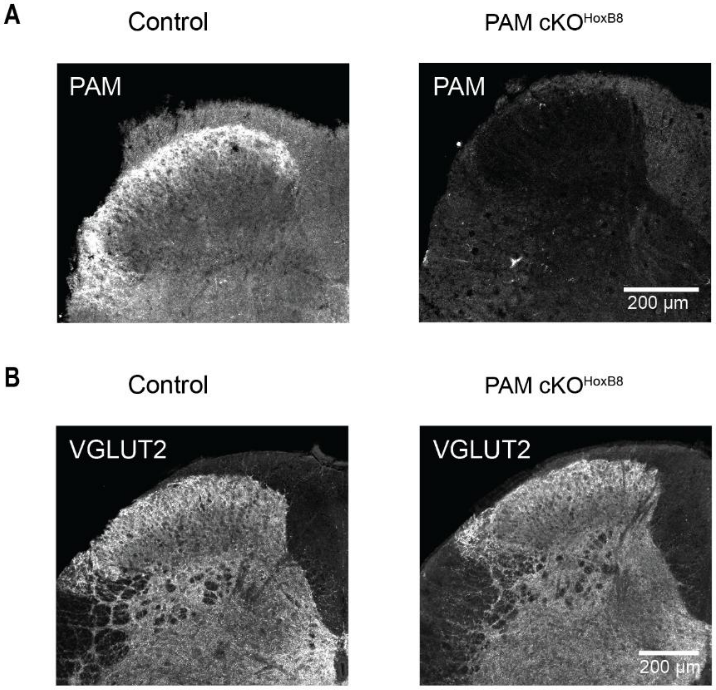

Fig. S3. Immunohistochemical staining of PAM cKO^{Hoxb8} dorsal horn. (A) Example confocal image showing that PAM is enriched in the superficial laminae of the dorsal horn of control mice (left). This staining is completely absent in the PAM cKO^{Hoxb8} (right). Note the overall reduction in staining intensity due to PAM deletion in all spinal cord cells in this mouse. **(B)** Example confocal image showing that VGLUT2 staining is similar in control and PAM cKO^{Hoxb8} dorsal horn.

bioRxiv preprint doi: https://doi.org/10.1101/2025.09.09.675093; this version posted September 11, 2025. The copyright holder for this preprint (which was not certified by peer review) is the author/funder. This article is a US Government work. It is not subject to copyright under 17 USC 105 and is also made available for use under a CC0 license.

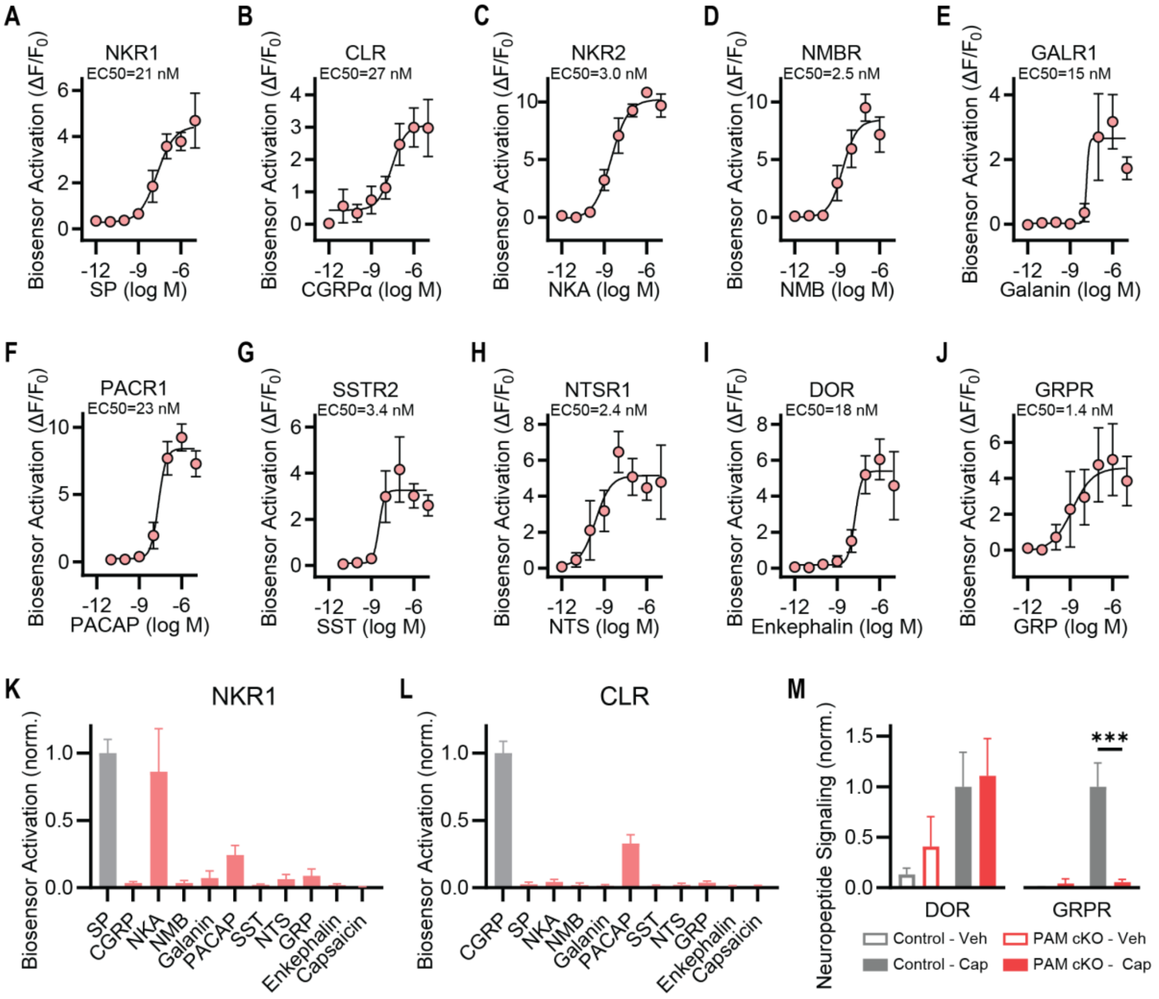

Fig. S4. Characterization of neuropeptide biosensors. (A-J) Dose response curves showing the sensitivity of each GPCR biosensor cell line to its target neuropeptide. Four-parameter variable slope dose-response curves were fit by non-linear regression. At least three replicates were acquired for each concentration, in two independent experiments. Please note the NKR1 and CLR data in (A) and (B) were previously published in Ref. (*18*), and are shown for comparison with the new cell lines. **(K)** Response of NKR1 cell line to a panel of neuropeptides. As expected, these cells are only activated by the tachykinins SP and NKA. **(L)** CLR cell lines specifically respond to SP. For (K) and (L), all neuropeptides were at 1 μM and capsaicin was at 10 μM. **(M)** Signaling by Enkephalin and GRP can be detected using DOR and GRPR biosensors from control nociceptors following capsaicin stimulation. PAM cKO nociceptors evoke normal DOR activity, but there is a loss of GRPR signaling. DOR: n=9 for Control; n=7 for PAM cKO. GRPR: n=10 for Control; n=13 for PAM cKO. Bars represent mean ± SEM. For each biosensor, means were compared using 2-way ANOVA followed by post-hoc Holm-Sidak's multiple comparison's test. *p<0.05, **p<0.01 ***p<0.001.

bioRxiv preprint doi: https://doi.org/10.1101/2025.09.09.675093; this version posted September 11, 2025. The copyright holder for this preprint (which was not certified by peer review) is the author/funder. This article is a US Government work. It is not subject to copyright under 17 USC 105 and is also made available for use under a CC0 license.

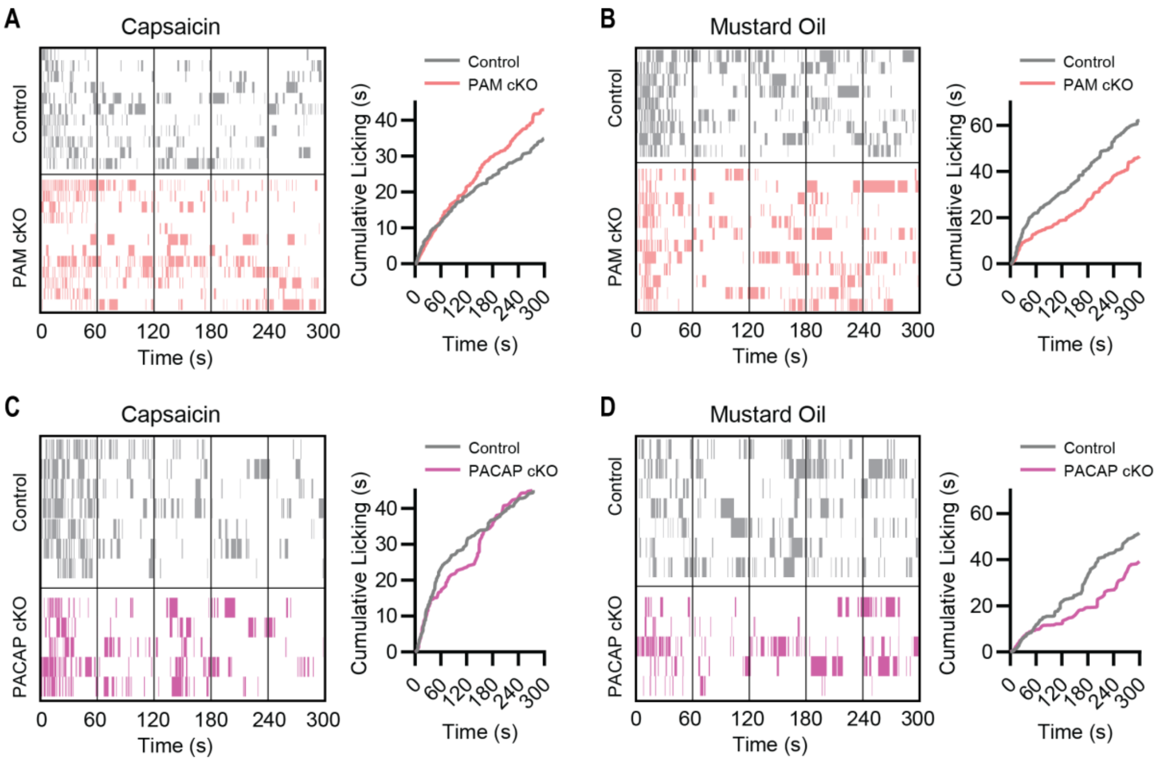

Figure S5. Temporal structure of chemical pain in PAM and PACAP cKO mice. (A-D) Ethograms and normalized cumulative licking plots for littermate control, PAM cKO and PACAP cKO mice given intraplantar injections of the chemical algogens capsaicin and mustard oil. (A) For capsaicin, n=12 (6M/6F) for Control and n=12 (8M/4F) for PAM cKO. (B) For mustard oil, n=9 (2M/7F) for Control and n=12 (3M/9F) for PAM cKO. (C) For capsaicin, n=7 (4M/3F) for Control and n=5 (3M/2F) for PACAP cKO. (D) For mustard oil, n=7 (4M/3F) for Control and n=5 (3M/2F) for PACAP cKO.

bioRxiv preprint doi: https://doi.org/10.1101/2025.09.09.675093; this version posted September 11, 2025. The copyright holder for this preprint (which was not certified by peer review) is the author/funder. This article is a US Government work. It is not subject to copyright under 17 USC 105 and is also made available for use under a CC0 license.

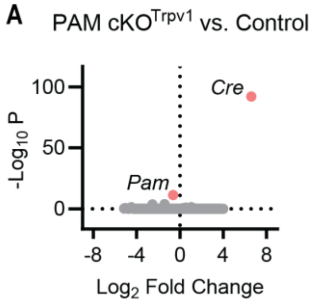

Figure S6. Deletion of PAM in Trpv1 afferents does not cause marked transcriptomic changes. (A) Bulk RNA sequencing shows very few genes are differentially expressed between control and PAM cKO dorsal root ganglia. As expected, Cre was enriched in PAM cKO^{Trpv1} DRGs, and PAM was downregulated. n=4 (2M/2F) for Control; n=4 (2M/2F) for PAM cKO^{Trpv1}.

bioRxiv preprint doi: https://doi.org/10.1101/2025.09.09.675093; this version posted September 11, 2025. The copyright holder for this preprint (which was not certified by peer review) is the author/funder. This article is a US Government work. It is not subject to copyright under 17 USC 105 and is also made available for use under a CC0 license.

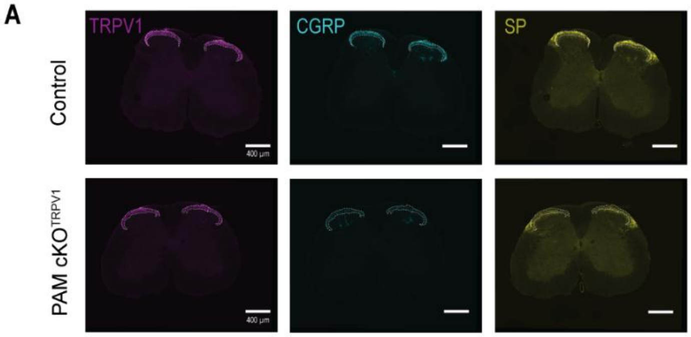

Figure S7. Deletion of PAM in Trpv1 afferents reduces neuropeptide staining. (A) Example confocal images of spinal cord dorsal horns showing that PAM deletion reduces the intensity of CGRP and Substance P (SP) staining in the terminal field of Trpv1 afferents. The dotted lines are traced from the region of Trpv1 staining. Note that only in the area of Trpv1 innervation would we expect to see reduced neuropeptide staining using the Trpv1-Cre approach.

bioRxiv preprint doi: https://doi.org/10.1101/2025.09.09.675093; this version posted September 11, 2025. The copyright holder for this preprint (which was not certified by peer review) is the author/funder. This article is a US Government work. It is not subject to copyright under 17 USC 105 and is also made available for use under a CC0 license.

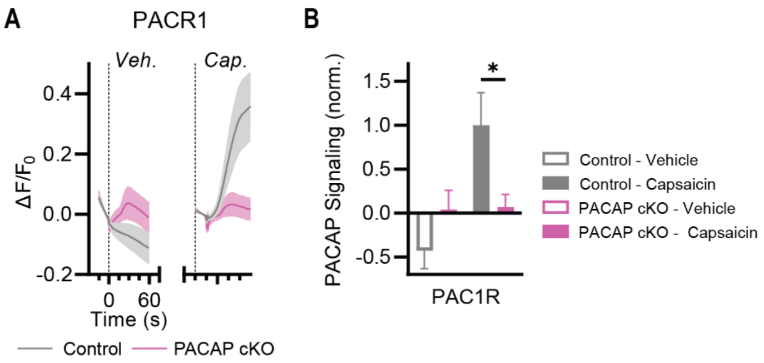

Figure S8. Loss of PACAP signaling from nociceptors in PACAP cKO mice. (A) Time course of PACAP release from nociceptors following capsaicin stimulation shows a complete loss in the PACAP cKO. **(B)** Summary plots showing PACAP signaling is abolished following PACAP deletion. n=13 from 2 Control mice; n=16 from 3 PACAP cKO mice. Bars represent mean ± SEM. Means were compared using 2-way ANOVA followed by post-hoc Holm-Sidak's multiple comparison's test. *p<0.05.

bioRxiv preprint doi: https://doi.org/10.1101/2025.09.09.675093; this version posted September 11, 2025. The copyright holder for this preprint (which was not certified by peer review) is the author/funder. This article is a US Government work. It is not subject to copyright under 17 USC 105 and is also made available for use under a CC0 license.

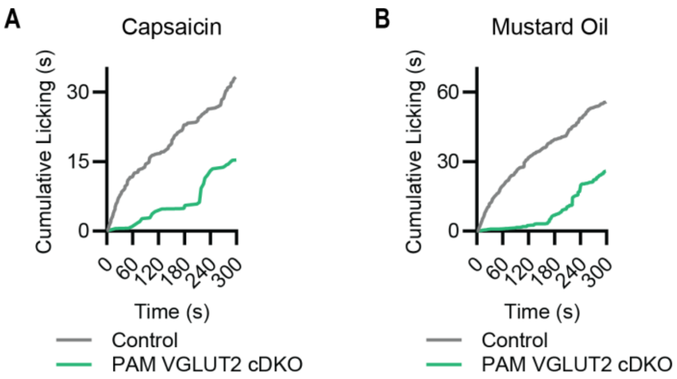

Figure S9. Temporal structure of chemical pain in PAM VGLUT2 cKO mice. (A) Cumulative licking (normalized to number of mice per group) to capsaicin. n=8 (5M/3F) for Control and n=6 (5M/1F) for PAM VGLUT2 cDKO. **(B)** Mustard oil-evoked cumulative licking. n=10 (5M/5F) for Control and n=12 (6M/6F) for PAM VGLUT2 cDKO.

bioRxiv preprint doi: https://doi.org/10.1101/2025.09.09.675093; this version posted September 11, 2025. The copyright holder for this preprint (which was not certified by peer review) is the author/funder. This article is a US Government work. It is not subject to copyright under 17 USC 105 and is also made available for use under a CC0 license.

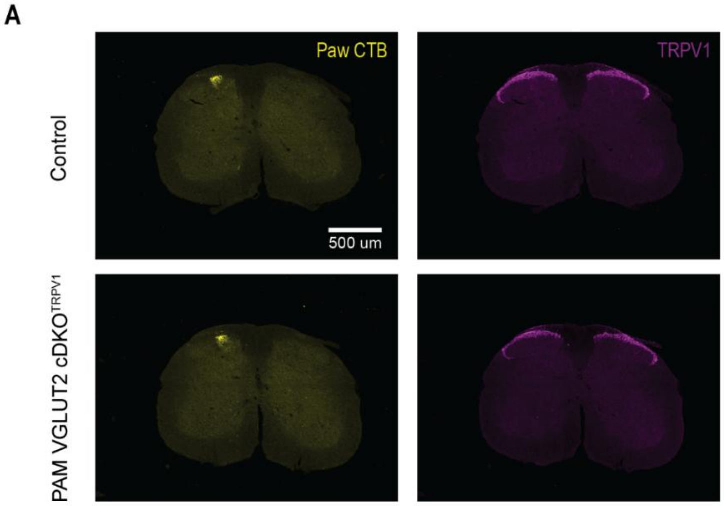

Figure S10. Combined deletion of PAM and VGLUT2 does not affect central terminal innervation. Example confocal images showing spinal cord dorsal horn staining for Trpv1 and cholera toxin B injected into the paw in Control (top) and PAM VGLUT2 cDKO mice (bottom).
